# Automatic Electric Tricycles Trajectory Tracking and Multi-Violation Detection

**DOI:** 10.3390/s25165135

**Published:** 2025-08-19

**Authors:** Leishan Guo, Bo Yu, Benhao Xie, Geng Zhao, Yuan Tian, Jianqing Wu

**Affiliations:** 1 School of Qilu Transportation, Shandong University, Jinan 250062, China; 202420944@mail.sdu.edu.cn (L.G.); benhao.xie@nottingham.edu.cn (B.X.); 2 Shandong Jinqu Design & Consulting Group Co., Ltd., Jianan 250014, China; boyu@163.com (B.Y.); zhaogeng@163.com (G.Z.)

**Keywords:** trajectory tracking, violation detection, target detection, electric tricycles

## Abstract

The escalating traffic violations associated with electric tricycles pose a critical challenge to urban traffic safety. It is important to automatically track the trajectories of electric tricycles and detect the multi-violations related to electric tricycles. This paper proposed an Electric Tricycle Object Detection (ETOD) model based on the custom-built dataset of electric tricycles. ETOD can successfully achieve real-time and accurate recognition and high-precision detection for electric tricycles. By integrating a multi-object tracking algorithm, an Electric Tricycle Violation Detection System (ETVDS) was developed. The ETVDS can detect and identify violations including speeding, passenger overloading, and illegal lane changes by plotting electric tricycle trajectories. The ETVDS can identify the conflicts related to electric tricycles in complex traffic scenarios. This work offers an effective technological solution for mitigating electric tricycle traffic violations in challenging urban environments.

## 1. Introduction

**Figure 1:**
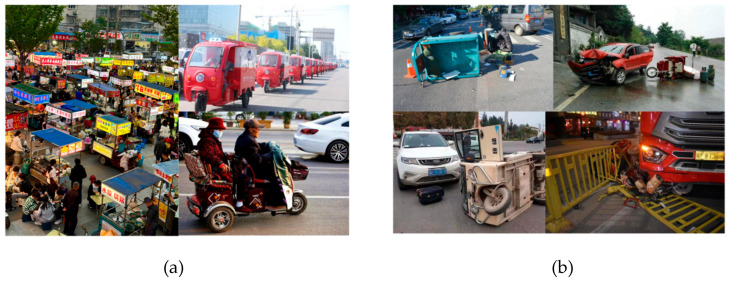
The widespread use of electric tricycles and associated violations. (**a**) Application of electric tricycles. (**b**) traffic accident.

Electric tricycles are an important transportation method due to their intrinsic advantages: agility, convenience, affordability, energy efficiency, and environmental friendliness. Electric tricycles can effectively cater to the mobility requirements of vulnerable populations, including the elderly and disabled [1,2] and prove invaluable in sectors such as courier logistics [3], as shown in Figure 1a. Nevertheless, the proliferation of electric tricycles has caused critical traffic safety concerns, such as speeding and overloading, as shown in Figure 1b. Trajectory tracking and conflict detection are important for electric tricycle management [4].

Many studies have been conducted for object detection for common entities such as sedan [5,6], motorcycles [7], non-motorized vehicles [8], pedestrian [9,10], road feature [11,12,13], and traffic sign [14,15]. However, there is a notable void in studies related to electric tricycles. Unlike automobiles, electric tricycles come in a wide variety of types, including cargo, passenger, sanitation, and specialized vehicles. These different models vary in their structure, configuration, performance standards, and other aspects, which increases the difficulty of inspection.

The core objective of violation detection is to maintain traffic order and safety. Violation detection systems monitor traffic violations in real-time, deterring potential offenders, and ensuring road safety [16,17,18]. Simultaneously, they enhance law enforcement efficiency and promote compliant driving behavior. Furthermore, they facilitate the collection of crucial data [19,20,21], supporting the optimization of traffic management [22,23], and improving traffic flow, ultimately contributing to a safe and efficient road traffic environment. Despite these benefits, a significant gap persists in the application of violation detection to electric tricycles. Given the increasing importance of electric tricycles in the traffic environment, and the challenges posed by their diverse range of models, bridging this gap by developing violation detection technology specifically for electric tricycles has become a pressing and critical research area. This necessitates dedicated research efforts and specialized technical solutions.

This study presents an innovative intelligent supervision method for detecting electric tricycle traffic violations. Our primary contribution lies in the development of a two-fold approach: First, an electric tricycle object detection (ETOD) model was developed. For performance optimization, EfficientNet was used as the backbone network, achieving a balance between computational efficiency and accuracy. To improve small target detection, an extra detection head was added, significantly enhancing the recognition accuracy and robustness for electric tricycles and their occupants in complex environments. A sophisticated trajectory tracking algorithm was integrated with a multi-object association analysis model to form an advanced Electric Tricycle Violation Detection System (ETVDS). This integrated system directly empowers the accurate identification and monitoring of electric tricycle violations, offering a crucial tool for enhancing traffic order and safety.

## 2. Related Work

This section reviews related works from two perspectives: object detection and trajectory tracking.

### 2.1. Object Detection

Traditional object detection algorithms primarily rely on manually designed filter features. Those approaches typically involve extracting candidate boxes using a sliding window method, followed by feature extraction and classification. Non-maximum suppression (NMS) is employed to merge the candidate boxes, eliminating overlapping or redundant boxes to output the final results. Typical algorithms include V-J [24] (Viola-Jones), HOG + SVM [25], and DPM algorithms [26], among others.

The core concept of two-stage object detection algorithms is to divide the object detection task into two phases: first, generating region proposals (candidate regions), which can be produced through selective search or anchor-based methods, and then classifying these candidate regions and performing bounding box regression. Representative algorithms of this approach include the R-CNN series (R-CNN [27], Fast R-CNN [28], Faster R-CNN [29], and Mask R-CNN [30]).

In contrast, single-stage object detection algorithms integrate candidate region generation and object classification within a single network, offering higher detection speed. Single-stage detection algorithms can directly output detection classifications and box boundaries prediction successfully through a single processing pass. Therefore, these detection algorithms exhibit good detection speed and are suitable for mobile devices, while leaving sufficient structural space for adding algorithm modules to accommodate various application needs. Single-stage object detection algorithms can be categorized into four types: the entire YOLO (You Only Look Once) series [31], SSD [32], RetinaNet [33], and EfficientDet [34]. Since its inception, YOLO has developed to YOLOv11, with continuous improvements in its network structure and performance.

### 2.2. Trajectory Tracking

Trajectory tracking is mainly classified into two categories: single object tracking (SOT) and multi-object tracking (MOT) [35]. One of the main drawbacks of SOT algorithms is the ambiguity in associating detected objects with their corresponding trajectories. This ambiguity can lead to identity switches (incorrect tracking) due to multiple objects being physically close together temporarily, resulting in overlapping spatial measurements [36]. MOT algorithms address identity switching by employing data association to match detected objects with their respective trajectories, utilizing methods such as the Monte Carlo algorithm [37], multiple hypothesis tracking (MHT) [38], and joint probabilistic data association (JPDA) [39]. However, these algorithms are computationally complex and resource-intensive.

In 2016, A. Bewley et al. introduced the simple online and real-time tracker (SORT) algorithm, which uses a Kalman filter to track trajectories and a Hungarian algorithm for data association between frames [40]. This method matches targets in the current frame with tracked targets in previous frames by minimizing the association cost, thereby determining the identity and trajectory of the targets. The SORT algorithm offers accuracy and robustness comparable to other MOT algorithms while being computationally efficient and faster. SORT can effectively track targets with limited resources but faces challenges due to non-linear camera motion and re-identification. Wojke et al.’s DeepSORT [41] offers improvements by addressing these weaknesses. It introduces a deep learning model to extract appearance features of targets for nearest-neighbor matching during real-time object tracking, using recursive Kalman filtering and frame-by-frame Hungarian data association. This allows the tracker to maintain object tracking over longer occlusion periods while remaining lightweight and maintaining real-time capabilities [42].

To summarize, an intelligent traffic detection system effectively addresses the management challenges posed by electric tricycles through the strategic integration of object detection technologies for their precise identification and localization, complemented by multi-object tracking techniques that enable real-time monitoring of their dynamic behaviors and robust handling of occlusion challenges. This holistic approach presents an efficient and reliable solution. Consequently, the imperative to develop video-based recognition and tracking technologies specifically tailored for electric tricycles cannot be overstated; it is not merely of significant practical importance but a fundamental necessity for achieving enhanced traffic management efficiency and improved road safety.

## 3. Methodology

**Figure 2:**
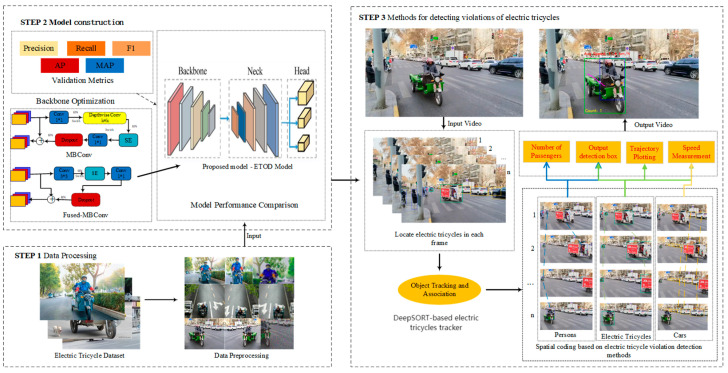
Structural framework diagram.

The ETOD model, trained on a custom-built electric tricycle dataset, is employed in this study. Subsequently, a multi-object tracking algorithm was integrated to create the ETVDS, enabling functionalities including electric tricycle recognition, trajectory tracking, and detecting passengers. A detailed schematic of the ETVDS is presented in Figure 2.

### 3.1. Model Construction

**Figure 3:**
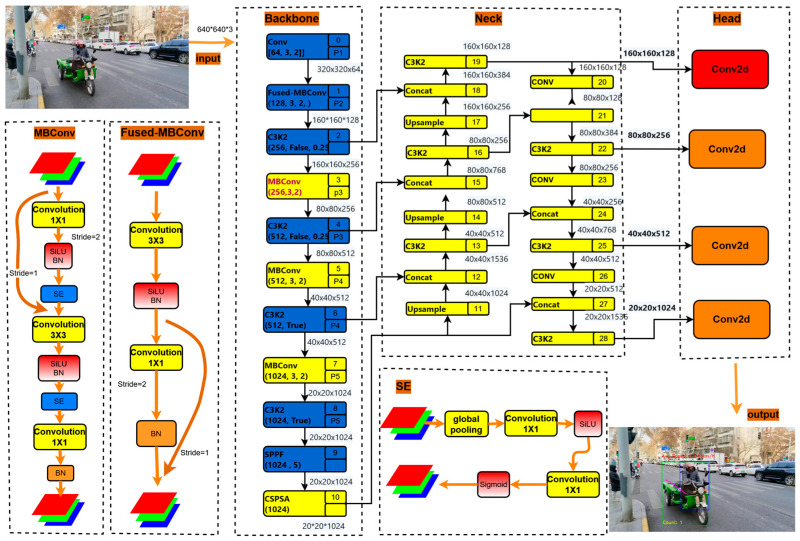
ETOD structural diagram.

Electric tricycles, valued for their compact dimensions and maneuverability, are nevertheless prone to occlusions in intricate urban traffic environments, thus requiring high-performance object detection. To meet these demands for electric tricycle detection in complex scenes, we optimized the model by integrating a dedicated small object detection head to improve detection accuracy. Moreover, the adoption of EfficientNet as the backbone further enhances the model’s detection efficiency and accuracy. A schematic representation of the model architecture is provided in Figure 3.

The primary improvements implemented in our model are as follows:

(1) Lightweight design: Achieving a lightweight object detection model is crucial for enhancing computational efficiency and optimizing resource utilization. This is accomplished by refining the network architecture and parameter configuration, which significantly reduces computational complexity while preserving detection performance, ultimately leading to faster inference speeds [43]. In this study, EfficientNet was selected as the backbone of our model. EfficientNet is renowned for its efficient network architecture, primarily due to its advanced design and training methodologies. By employing automatic network architecture search and compound scaling strategies, EfficientNet effectively minimizes computational load and parameter count while maintaining high accuracy, thereby facilitating high efficiency inference across diverse devices [44].

Our model incorporates EfficientNetV2 as its backbone. EfficientNetV2, the second generation in the EfficientNet series, was initially presented by Google researchers at the 2021 ICML conference. This advanced iteration significantly improves the trade-off inherent in computational efficiency and model accuracy by combining Fused-MBConv blocks, an optimized neural architecture search, and progressive learning strategies [45]. A notable improvement in EfficientNetV2 is the ingenious design of Fused-MBConv. This addresses the bottleneck caused by the slow execution of depth-wise convolution (DWConv) within the MBConv modules of deeper networks, primarily due to insufficient hardware acceleration support. Conceptually, Fused-MBConv merges the 1 × 1 point-wise and DWConv operations of the MBConv module into a single, efficient 3 × 3 standard convolution (as visually represented in Figure 3). Critically, this fusion strategy is most effective when applied predominantly in the initial stages of the network, rather than as a complete replacement throughout all layers. Based on thorough experimental comparisons, this paper ultimately adopts the ETOD version, with its precise structural configuration detailed in Figure 3.

(2) Addressing small object detection challenges: In road surveillance imagery, electric tricycles inherently present as small-scale objects relative to other common detection targets. Conventional multi-scale feature fusion strategies, commonly employed in most object detection architectures, often prove inadequate for the precise localization of such small objects [46]. Standard configurations typically incorporate three detection heads operating on feature maps of sizes 80 × 80, 40 × 40, and 20 × 20, designed to detect objects of approximate scales 8 × 8, 16 × 16, and 32 × 32 pixels or larger, respectively. Nevertheless, the intrinsic detection capability for truly tiny objects remains a significant challenge. To mitigate this, we augmented the model with an additional detection head by leveraging a new 160 × 160 feature map. This new head is specifically configured to identify targets as small as 4 × 4 pixels, thereby substantially bolstering the model’s performance on small object detection. The integration point of this novel detection head is visually represented as the red component in Figure 3.

### 3.2. Multi-Object Tracking

The core of multiple-object tracking (MOT) algorithms centers on the integration of target appearance features and motion cues, along with optimized target matching strategies. By combining detection outputs with techniques such as the Hungarian algorithm and Kalman filtering, the system enables continuous and robust tracking of multiple targets. Specifically, the MOT process involves three primary steps: (1) target detection and feature extraction; (2) data association, utilizing the Hungarian algorithm to establish optimal matches and maintain track trajectories; and (3) trajectory management. The overall workflow is summarized in Algorithm 1.
**Algorithm 1: Multiple-Object Tracking Algorithm**Input:      tracks: List of Track objects (initially empty)      detection_list: List of Detection objects in the current frame Initialization:      for each detection in detection_list:            track = Create_Track(detection.bounding_box)  // Create a new Track object            track.state = ‘unconfirmed’            Append track to tracks Processing each frame:      1. Prediction step:            for each track in tracks:                   track.predicted_state = Kalman_Predict(track.state)  // Kalman filter prediction                   track.predicted_box = Project_to_Image(track.predicted_state)      2. Cost matrix calculation:            cost_matrix = Create_Cost_Matrix(tracks, detection_list)            /*Cost_Matrix(i, j) = Cost of associating track i with detection j */      3. Data association:            (matched_tracks, unmatched_tracks, unmatched_detections) = Hungarian_Algorithm(cost_matrix, tracks, detection_list)            /*            matched_tracks: List of (track_index, detection_index) tuples            unmatched_tracks: List of track indices            unmatched_detections: List of detection indices            */      4. Update tracks:            // Update matched tracks            for (track_index, detection_index) in matched_tracks:                   track = tracks[track_index]                   detection = detection_list[detection_index]                   Update_Track(track, detection)            // Handle unmatched tracks            for track_index in unmatched_tracks:                   track = tracks[track_index]                   if track.state == ‘unconfirmed’:                         // Delete unconfirmed tracks                         Remove track from tracks                   else:                         track.age += 1 // Increment age for potential deletion later                         if track.age > max_age:        //max_age = 30 frames                               Remove track from tracks            // Handle unmatched detections            for detection_index in unmatched_detections:                   detection = detection_list[detection_index]                   track = Create_Track(detection.bounding_box)                   track.state = ‘unconfirmed’                   Append track to tracks      5. State update:            for track in tracks:                   if track.state == ‘confirmed’ or track.age < min_hits:   //min_hits = 3 frames                         track.state = ‘confirmed’   // Tracks must be matched for min_hits before confirmation                   else:                         track.state = ‘tentative’      // Marks as temporary before removal Output:      Return tracks  // List of all Track objects (containing confirmed, tentative, and unconfirmed tracks)

### 3.3. Violation Detection Methods

This study develops an Electric Tricycle Vehicle Detection System (ETVDS) that combines an ETOD model with multi-object tracking to achieve high-accuracy, real-time detection and tracking of electric tricycles. The ETVDS, operating in complex traffic environments, enables the detection of traffic violations such as speeding and overloading via functionalities like passenger counting, trajectory plotting, and speed measurement.

#### 3.3.1. Passenger Counting Function

The passenger counting methodology primarily relies on the ETOD model’s real-time object detection capacity for electric tricycles and pedestrians. The initial step involves detecting all electric tricycles and rendering their respective bounding boxes. To accurately identify passengers, a hierarchical thresholding scheme based on intersection over union (IoU) is then applied:

(1) Spatial overlap assessment (*CIOU*): A “co-occurrence IoU“ (*CIOU*) threshold is defined to quantify the degree of spatial overlap between the detection boxes of electric tricycles and pedestrians. This serves as a primary filter for potential passenger candidates.
(2) Positional proximity tracking (*WIOU*): Following the *CIOU* filtering, a robust multi-object tracking algorithm is utilized to continuously monitor and compare the relative spatial positions of pedestrians and electric tricycles. A “wound IoU“ (*WIOU*) threshold is then established, which rigorously evaluates their sustained positional proximity, indicating a consistent physical relationship.
(3) Temporal consistency verification (*TIOU*): The final criterion is a “temporal IoU“ (TIOU) threshold. This critical threshold is met only when the *CIOU* and *WIOU* conditions are continuously satisfied for a predefined duration, thereby confirming a stable and prolonged association between a pedestrian and an electric tricycle.

A pedestrian is registered as a passenger and their bounding box displayed for counting only if all three IoU thresholds—*CIOU*, *WIOU*, and *TIOU*—are concurrently fulfilled. The mathematical formulation governing this process is presented subsequently.

Based on the ETOD model for object detection, the positions and categories of targets are identified, with each target’s bounding box represented by the center coordinates (*x*, *y*), width *w*, and height *h*. The duplication rate of the detection boxes for electric tricycles and pedestrians is as follows:

(1) $$CIOU=\frac{\left(w_{p}\times h_{p}\right)\cap \left(w_{\mathrm{vt}}\times h_{\mathrm{vt}}\right)}{w_{p}\times h_{p}}$$

In the formula, *CIOU* is represented as the duplicate *IOU*; $w_{p}$ is the width of the pedestrian’s bounding box; $h_{p}$ is the height of the pedestrian’s bounding box; $w_{\mathrm{vt}}$ is the width of the electric tricycle’s bounding box; and $h_{\mathrm{vt}}$ is the height of the electric tricycle’s bounding box.

(2) $$WIOU=\sqrt{{\left(x_{p}^{t}-x_{d}^{t}\right)}^{2}+{\left(y_{p}^{t}-y_{d}^{t}\right)}^{2}}$$

where *WIOU* is represented as position *IOU*; (*x*, *y*) are the center coordinates of the bounding box; p represents the pedestrian; and d represents the electric tricycle.

#### 3.3.2. Trajectory Plotting Function

Based on multi-object tracking algorithms [47], the trajectory management function enables the retrieval and display of electric tricycle trajectory information.

Using the ETOD model, obtain the position information of each target, including the center coordinates (*x*, *y*). Apply the multi-object tracking algorithm to track the detected targets, with each detected target being assigned a unique ID, as shown in the following formula.

(3) $${Box}_{1}=(x_{1},y_{1})$$

In the formula, *Box* represents a set; 1 is the unique ID information of the target; and (*x*_1_*, y*_1_) are the center coordinates of the target.

Additionally, the position information of each target is stored for subsequent tracking, as shown in the following formula:

(4) $$\begin{array}{c} {\left(T\mathrm{rack}\_history\right)}^{1}=\left\{\left.Box_{1},Box_{2},\ldots Box_{m}\right\}\right.\\ •\\ •\\ •\\ {\left(T\mathrm{rack}\_history\right)}^{n}=\left\{\left.Box_{1},Box_{2},\ldots Box_{m}\right\}\right. \end{array}$$

In the formula, *m* represents the number of targets, and *n* represents the different frames of the video.

The historical position information of each target is extracted, as shown in the following formula:

(5) $$\begin{array}{c} {\left(T\mathrm{rack}\_history\right)}_{1}=\left\{\left.Box_{1}^{1},Box_{1}^{2},\ldots Box_{1}^{n}\right\}\right.\\ •\\ •\\ •\\ {\left(T\mathrm{rack}\_history\right)}_{m}=\left\{\left.Box_{m}^{1},Box_{m}^{2},\ldots Box_{m}^{n}\right\}\right. \end{array}$$

Based on the historical position information of each target, the trajectory of the target is drawn.

#### 3.3.3. Speed Calculation Function

Based on the target detection capability of the ETOD model, the length or width data of known targets is set [48], and a precise conversion between pixel coordinates and world coordinates is performed based on the pixel data of the detected targets in the video.

First, for each common vehicle ID, the pixel distance between two frames is calculated. This distance is obtained using the Euclidean distance formula, as shown in the following equation:

(6) $$\mathrm{pixel}\_\mathrm{distance}=\sqrt{{\left(x_{2}-x_{1}\right)}^{2}+{\left(y_{2}-y_{1}\right)}^{2}}$$

Obtain the actual width of the current target and the detected width. Since the model is applicable to roadside monitoring systems, most cameras are fixed cameras. Therefore, the actual width (real_width) is set based on real-world conditions, while the detected width (line_width) is obtained from the current frame data. The speed is calculated using the following formula (in km/h).

(7) $$\mathrm{speed}\_\mathrm{kmh}=\left(\frac{\mathrm{pixel}\_\mathrm{distance}\times \mathrm{real}\_\mathrm{width}}{\mathrm{line}\_\mathrm{width}}\right)\times \mathrm{fps}\times 3.6$$

This model not only effectively identifies and addresses violations by electric tricycles but also provides valuable data support for future traffic management. As research continues to deepen, this model is expected to play an increasingly important role in future traffic management.

## 4. Data Collection and Processing

### 4.1. Data Collection

Effective model training critically depends on robust data collection, which directly determines the resultant model’s performance. In this study, we constructed a comprehensive and representative dataset through a tripartite collection strategy, meticulously designed to capture the unique traffic dynamics pertinent to our research. The three primary data sources include (1) web scraping, (2) real-time surveillance footage acquired from existing urban intersection cameras, and (3) bespoke on-site data captured by the research team. These sources are further detailed in Table 1.

**Figure 4:**
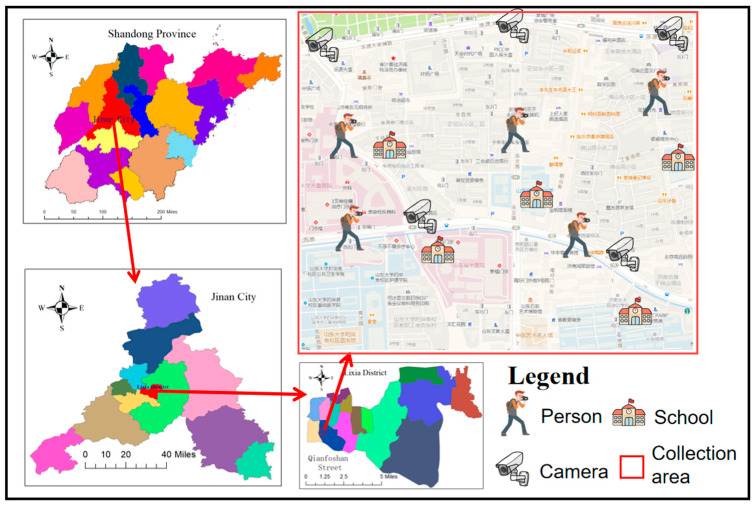
Data collection distribution map.

This study’s data collection focused on Lixia District, Jinan City, which is geographically bounded by Jingshi Road to the north, Qingnian East Road and Luowen Road to the west, Shunhe Elevated Road to the east, and Luoyuan Street to the south. This specific area was prioritized for data acquisition due to its demographic characteristics, notably encompassing three schools and exhibiting a high population density. Such attributes contribute to a pronounced use of electric tricycles for child transport, rendering this region highly representative for our research objectives. A more comprehensive visualization of this critical data collection area is provided in Figure 4.

The first component of our multi-source strategy involved web-scraped data acquisition. Images specifically featuring electric tricycles were acquired via the Baidu search engine, leveraging keywords such as “electric tricycle,” “tricycle,” and “electric vehicle.” To broaden the contextual diversity of the dataset, images of cars and pedestrians from the COCO dataset were subsequently incorporated. This combination yielded a substantial number of high-resolution, diverse traffic scene images featuring electric tricycles alongside other relevant objects, including various vehicles, pedestrians, and traffic lights. These richly annotated scenes are inherently well-suited for deep learning model training. However, it is crucial to acknowledge the inherent limitations of web-scraped data, including potential copyright infringements and inconsistencies in data quality. Consequently, strict adherence to all applicable legal regulations and ethical guidelines was maintained throughout this data collection process.

As the second data source, real-time video streams from urban intersection cameras were accessed, specifically focusing on five designated intersections within the study area. Traffic events of interest were meticulously identified and manually segmented from these continuous video streams. Subsequently, individual frames were programmatically extracted using Python (v3.12), resulting in a static image dataset enriched with diverse traffic signs, vehicles, and pedestrian activity. Before further processing, all camera data underwent stringent standardization to enhance data integrity and protect sensitive information. This preprocessing included denoising techniques and brightness normalization, ensuring data consistency while mitigating potential privacy concerns.To ensure the privacy of all data, facial blurring has been applied to data containing individuals. 

Finally, recognizing a potential data deficit and the need for scene specificity, supplemental on-site data were collected through dedicated filming by the project team directly within the defined area of interest. This independent capture of real-world traffic conditions aimed to provide data reflecting the unique characteristics of the target environment, thereby enriching the model with diverse and localized scene information. To maximize the model’s generalization capabilities, significant emphasis was placed on ensuring the representativeness of the filmed scenarios, carefully avoiding the introduction of artificial effects or biases in the spatiotemporal distribution of the collected data. This comprehensive on-site data collection process is also illustrated in Figure 4.

### 4.2. Data Processing

The initial phase of our methodology involved systematic data acquisition and comprehensive preprocessing. This crucial step aimed to significantly enhance both the quality and diversity of the collected data, a critical prerequisite for achieving effective model training and rigorous testing.

Data cleaning was the first priority, meticulously removing blurry, redundant, and irrelevant images to ensure the dataset’s fidelity in capturing the intricate nature of the research subjects. This was followed by essential data preprocessing and thorough quality assurance, which included standardized resizing, consistent format conversion, and the careful exclusion of any distorted images. Concurrently, a structured basic information database was developed to efficiently catalog and manage the dataset’s metadata.

The data annotation and segmentation phase commenced. Target objects within the images were meticulously labeled using LabelImg software(v1.3.1), precisely specifying their locations and categories. This detailed annotation was vital for ensuring the data’s usability and suitability for subsequent model training. The labeling task was executed by a dedicated four-member team, employing a rigorous two-step process: an initial annotation by one subgroup, followed by a thorough verification by another, thereby ensuring high data quality and consistency. Upon completion of annotation, the dataset was strategically partitioned into distinct training, validation, and testing sets to guarantee fairness and representativeness, and to facilitate unbiased model evaluation.

**Figure 5:**
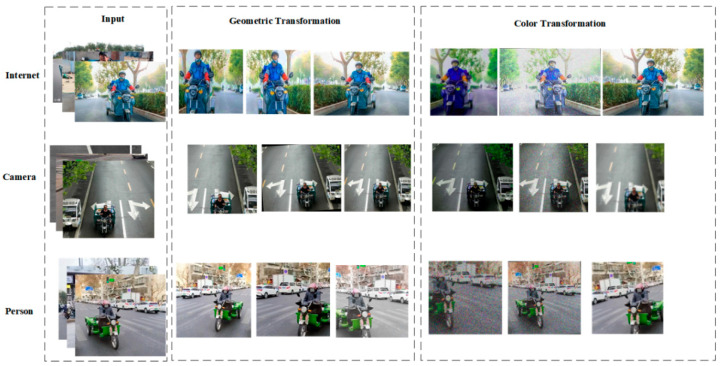
Data collection and processing.

To further enrich the training dataset’s variability and representativeness, data augmentation techniques were extensively applied. This involved implementing various geometric transformations, such as random cropping, rotation, and flipping, alongside colorimetric variations, including brightness adjustments. As illustrated in Figure 5, these synthetic expansions of the training data are designed to bolster the model’s generalization capabilities.

**Figure 6:**
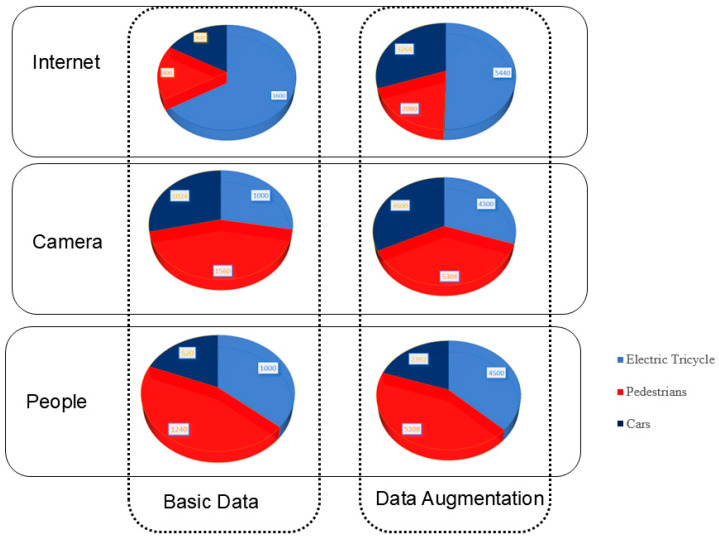
A visualization of the dataset before and after data augmentation.

This comprehensive suite of data processing and optimization methods not only ensures the dataset’s integrity and uniformity but also establishes a stable and robust platform for both model training and validation, ultimately contributing to the improved robustness and generalization ability of the resulting predictive model. The overall outcomes of this multi-faceted data processing pipeline are visually summarized in Figure 6.

## 5. Results

### 5.1. Model Training and Testing

We meticulously collected and organized a high-quality dataset comprising electric tricycles, pedestrians, and various vehicles. This comprehensive dataset was subsequently partitioned into a training set (70%) and a test set (30%).

For model training, we selected several representative object detection models known for their distinct strengths in optimizing detection performance from various perspectives. Specifically, chosen architectures included FCOS, EfficientDet, RT-DETR, YOLOv8,, and our improved ETOD model. FCOS and EfficientDet were chosen for their high detection accuracy in complex scenarios, while RT-DETR and YOLOv8 are recognized for their superior real-time detection capabilities. The ETOD model, a modified version tailored for electric tricycle detection, was specifically designed to further enhance detection efficacy for this task.

Each model underwent 100 epochs of training, with a batch size of 32 images. All image samples were uniformly resized to a resolution of 640 × 640 pixels. To mitigate overfitting and optimize model performance, a stringent early stopping strategy was implemented: training was terminated prematurely if validation metrics showed no improvement for 20 consecutive epochs. Upon the completion of training, the models were comprehensively evaluated using standard metrics such as precision, recall, and mean average precision (mAP) to thoroughly assess their performance.The specific parameters are as shown in Table 2.

### 5.2. Model Performance Verification

**Figure 7:**
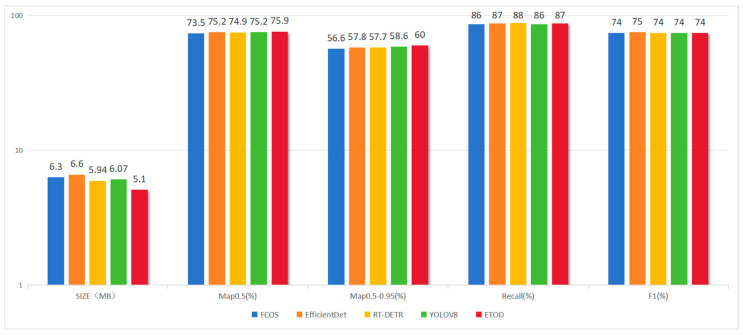
Comparison of Results for Each Model.

The models were trained using the curated dataset, and comparative performance is summarized in Figure 7. Notably, the modified ETOD model demonstrates superior accuracy, achieving this performance with a remarkably compact size of only 5.1 MB. This reduced model size offers substantial advantages in terms of resource efficiency.

**Figure 8:**
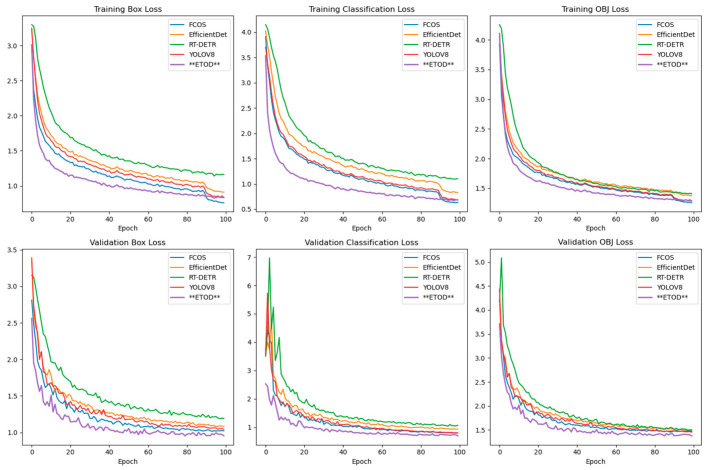
Comparison of Loss Results for Each Model During Training and Validation Phases.

As evidenced by the linear trends in Figure 8, the box loss, classification loss, and distribution focal loss consistently decrease throughout both the training and validation phases. These reductions correlate with improved accuracy in bounding box prediction, object classification, and confidence score estimation. Notably, the ETOD model demonstrates a steeper decline, suggesting superior convergence and potentially higher performance compared to the other models.

**Figure 9:**
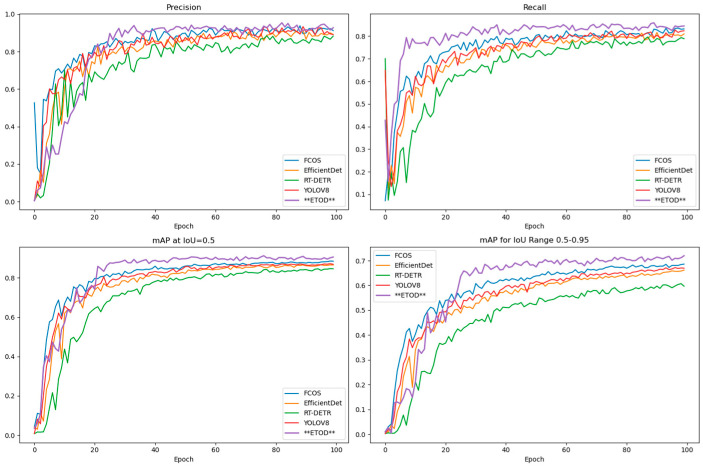
Comparison of results of various model indicators.

Although the accuracy of individual models exhibits some fluctuation, the overall trend indicates a gradual improvement in the ability to recognize vehicles. The recall rate, while variable, also generally trends upward, suggesting an enhanced capability to consistently detect targets. Notably, the mean average precision (mAP) at IoU thresholds of 0.50 (mAP@50) and 0.50–0.95 (mAP@50–95) demonstrates significant improvement throughout the training duration, confirming a substantial increase in both the accuracy and reliability of the models. The ETOD model exhibits a greater initial rate of improvement compared to the other models, although its rate diminishes in later stages, converging towards the performance levels of the others. However, as evidenced by the metrics presented in Figure 9 the overall performance of the ETOD model remains significantly superior. This superior performance, combined with its faster initial training gains, justifies the selection of the ETOD model for electric tricycle object detection in this research.

**Figure 10:**
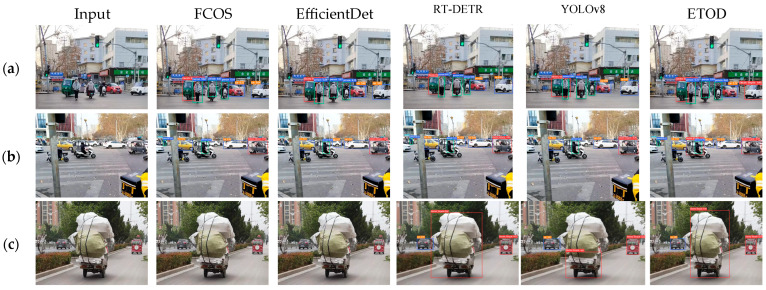
Detection effect of algorithm model in different scenarios. (**a**) irregular electric tricycle detection, (**b**) detection in complex occlusions, and (**c**) detection in load-carrying scenarios.

Motivated by practical application needs, this paper conducts detection experiments in three representative scenarios, with results presented in Figure 10. The comparison of detection results between the ETOD model and other algorithms reveals that ETOD achieves more accurate object recognition of irregular electric tricycles. In complex occlusion scenarios, ETOD also exhibits higher detection accuracy. Notably, in load-carrying scenarios, the detection performance of ETOD significantly surpasses that of comparative methods such as FCOS, EfficientDet, and RT-DETR. These results demonstrate the superior performance of the proposed ETOD model for electric tricycle detection, particularly exhibiting increased robustness and reliability in challenging real-world conditions.

Comparing the base model and the ETOD model, in terms of model performance, the ETOD model significantly reduces the model size (only 5.1 MB) while maintaining high accuracy, and demonstrates faster convergence speed and more stable performance improvement during training. The ETOD model demonstrates exceptional performance in electric tricycle detection, boasting superior accuracy and a lower false negative rate. Even in complex occlusion scenarios, the ETOD model excels, effectively identifying partially obscured targets. These results indicate that the ETOD model possesses stronger robustness and reliability when handling complex scenarios commonly encountered in real-world applications, providing an efficient and reliable solution for intelligent electric tricycle detection.

### 5.3. Model Detection Results Validation

**Figure 11:**
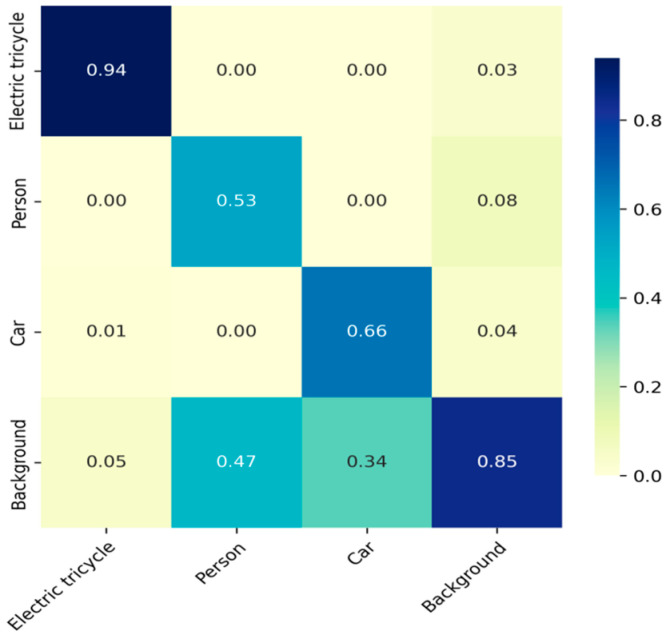
Confusion Matrix Results of Model Training.

The model’s precision was assessed through a confusion matrix. The precision for electric tricycles reaches 0.94, indicating that 94% of instances in this category are accurately identified, with only 6% being false positives classified as background. In contrast, the precision for pedestrians and cars is only 53% and 66%, respectively. This is primarily due to the lower accuracy of the COCO dataset for these other models [49]. Overall, the model demonstrates good precision.As shown in Figure 11. 

**Figure 12:**
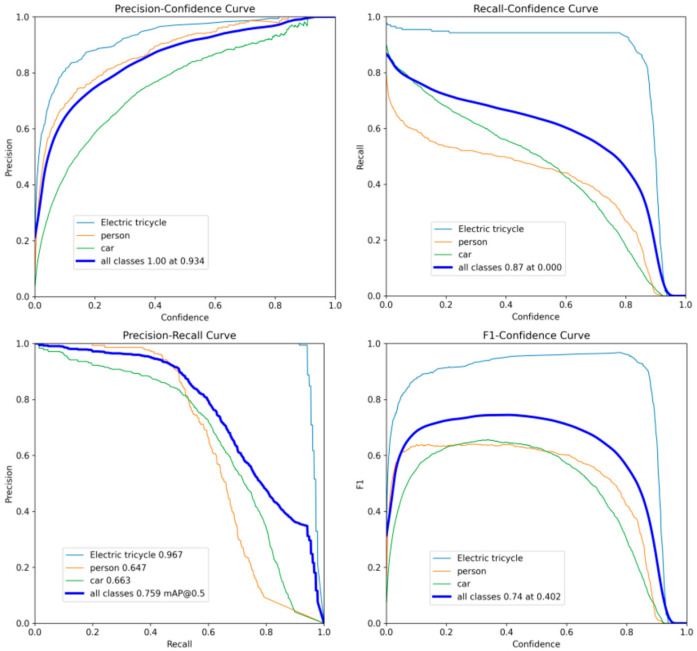
Results of model training for precision, recall, and average precision.

Figure 12 effectively illustrates the intricate relationship between precision, recall, and the specified confidence thresholds. A particularly salient finding is the attainment of a perfect precision score (1.00) when the confidence threshold surpasses 0.934. This signifies that, at such a stringent threshold, all positive predictions made by the model are unequivocally accurate, indicating the complete absence of false positives.

Conversely, recall performance exhibits substantial variability with the manipulation of the confidence threshold. Intriguingly, recall is observed to be 0.000 when the confidence threshold is less than 0.87. However, precisely at a threshold of 0.87, the model achieves a perfect recall score of 1.00. This remarkable performance at this specific point underscores the model’s capability to correctly identify and retrieve all actual target instances.

The precision–recall (PR) curve (implicitly, if displayed as in Figure 12) is instrumental in elucidating the inherent trade-off between precision and recall: typically, a pursuit of higher recall may lead to a decrease in precision, and vice versa. A PR curve that closely approaches the upper-right corner of the plot is indicative of superior model performance, demonstrating its capacity to achieve simultaneously high levels of both precision and recall. In this context, the presented PR curve affirms the model’s robust prediction capabilities, achieving an impressive mean average precision (mAP@0.5) of 0.759 across all object categories. This metric further accentuates the model’s excellent overall object detection efficacy.

Furthermore, the F1 curve serves as a comprehensive metric for evaluating the combined performance of precision and recall. The F1 score, calculated as the harmonic mean of these two metrics, ranges from zero to one. Notably, at a confidence threshold of 0.402, the model yields an F1 score of 0.74, which provides a holistic assessment of its detection accuracy and recall ability.

In conclusion, the model demonstrates exceptional proficiency in electric tricycle detection. The combined high precision of 94%, a respectable mAP@0.5 of 0.759, and a solid F1 score of 0.74 collectively affirm its outstanding object detection capabilities.

### 5.4. Multi-Target Tracking Verification

Based on the constructed ETOD model, we implemented multi-object tracking by integrating it with a multi-object tracking algorithm. It was evaluated using three traffic intersection detection videos, all filmed by researchers on traffic roads in Jinan, Shandong. The video information is shown in Table 3 below.

To validate the multi-object tracking performance of the model, team members manually collected the actual data of electric tricycles and other detected targets from the test videos. These data were then cross-verified with the number of detected targets and trajectories obtained from the code output. Vehicle detection performance and validation were further assessed using recall rate and precision metrics. The results are shown in Table 4.

The results show that the trajectory construction accuracy of electric tricycles reached over 90% in all three test videos. The accuracy for pedestrians and cars was relatively lower, which may be related to the relatively lower object recognition accuracy of the preceding ETOD model. The ETOD model demonstrates strong performance in multi-object tracking tasks, particularly excelling in the trajectory tracking of electric tricycles.

### 5.5. Violation Detection

**Figure 13:**
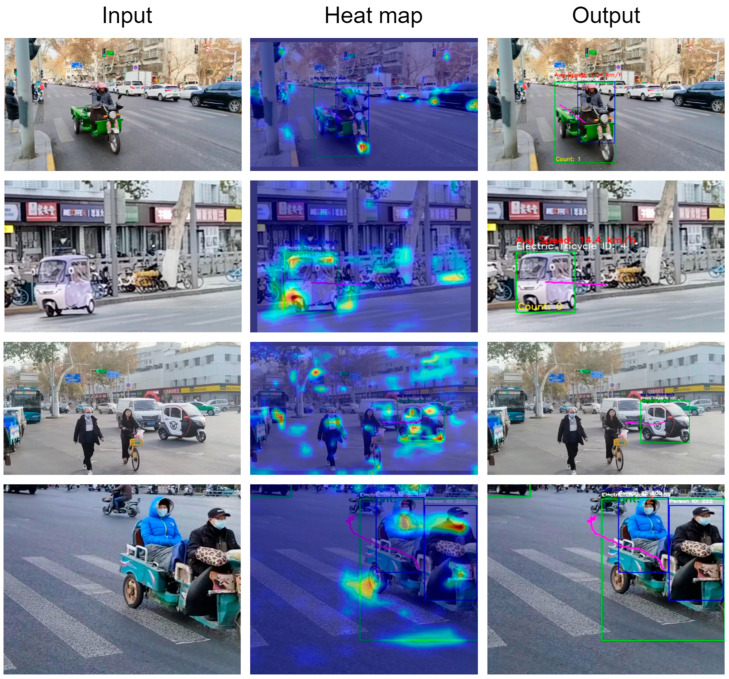
Model training and validation metrics results.

Leveraging a target detection model trained within the ETOD framework, in conjunction with multi-object tracking algorithms, we developed the Electric Tricycle Violation Detection System (ETVDS). The system encompasses methodologies for passenger counting, trajectory plotting, and speed determination. The results are shown in Figure 13.

Heatmaps offer an intuitive means of understanding the regions of an image most salient to the model’s decision-making process. By examining the heatmap, one can identify regions in the feature map exhibiting elevated activation values, thus gaining insight into the features to which the model directs its attention. As illustrated in Figure 13, for electric tricycle detection, the area surrounding the front wheel exhibits a high activation value, suggesting this region is a critical feature for accurate object identification.

The electric tricycle violation detection method demonstrates proficiency in basic object tracking and trajectory analysis. However, the accuracy of passenger counting is notably reduced. This is primarily due to limitations imposed by data acquisition during winter. The implementation of cold-weather protective measures, such as coverings, on electric tricycles significantly occludes the view of passengers. Furthermore, certain electric tricycle models exhibit structural elements that contribute to visual obstruction, thereby hindering the accuracy of the passenger counting method and leading to suboptimal passenger recognition.

## 6. Conclusions

This paper introduces ETOD (Enhanced Tracking and Detection for Electric Tricycles), a lightweight (5.1 MB) model designed for improved object detection and multi-object tracking, specifically targeting electric tricycle-related violations. ETOD demonstrates exceptional performance in electric tricycle detection, achieving a 94% accuracy rate, even in complex occlusion scenarios. By integrating a multi-object tracking algorithm, ETOD achieves over 90% accuracy in tracking electric tricycle trajectories, although tracking precision for pedestrians and other vehicles is comparatively lower. Building upon these advancements, this study successfully develops a violation detection system for electric tricycles, capable of tasks such as passenger counting (with minimal impact from occlusions), trajectory visualization, and speed measurement. This system offers an efficient solution for electric tricycle management and contributes to the development of smart cities and advanced transportation infrastructure.

While promising, the current research presents several limitations that future work will address. First, the current dataset is relatively small, representing specific environmental conditions with limited seasonal sample data, potentially impacting the model’s generalization capabilities. Future efforts will focus on expanding the dataset to encompass a wider range of "scenarios," further optimizing model performance under diverse conditions. Second, the current speed detection method relies on a known target length and operates effectively only at fixed angles. Future research will explore improved methodologies, incorporating techniques like depth estimation, to enhance accuracy and broaden the applicability of speed detection in more dynamic and variable environments. Finally, the model’s passenger counting functionality is relatively weak. Through the incorporation of algorithms such as pose estimation, continuous optimization of the ETOD model’s underlying structure and algorithms will be pursued to further enhance its detection accuracy and efficiency. These advancements will significantly contribute to the effective management of electric tricycles and the broader advancement of intelligent transportation systems.

## Figures and Tables

**Table 1:** Dataset source statistics table.

Method	Type	Category	Quantity	Source
Web	Image	Electric Tricycles	1600	Web
Web	Image	Pedestrians, Cars	2000	COCO Dataset
Traffic Surveillance Camera	Video	Electric Tricycles, Pedestrians, Cars	1.2 GB	Traffic Management Authorities
On-site Collection	Image	Electric Tricycles, Pedestrians	680	Data Collection Area

**Table 2:** Comparison of Results for Each Model.

Parameter Name	Parameter Value
Training Epochs	100
Batch Size	32
Image Resolution	640 × 640 pixels
Learning Rate	Initial learning rate: 0.001, with learning rate decay (e.g., decay by 0.1 every 10 epochs)
Early Stopping	Training terminates if validation metrics show no improvement for 20 consecutive epochs
Training Device	NVIDIA RTX4050
CUDA version	12.6
Data Loading Threads	8 threads
Model Saving Frequency	Save model weights every 5 epochs
Logging Frequency	Record training and validation metrics every epoch

**Table 3:** Test video information.

Information	Test Video #1	Test Video #2	Test Video #3
Road geometry	With intersection	Without intersection	Without intersection
Traffic condition	Free-flow	Congested	Free-flow
Frame rate	24 fps	24 fps	24 fps
Resolution	1920 × 1080	1920 × 1080	1920 × 1080

**Table 4:** Model trajectory construction performance.

Title 1	Test Video #1			Test Video #2			Test Video #3		
	ET	PT	CAR	ET	PT	CAR	ET	PT	CAR
Ground truth	35	1024	2256	61	842	1680	52	1427	2043
True positive	31	743	1654	53	643	1320	45	1023	1542
False negative	4	281	602	8	199	360	7	404	501
False positive	3	112	246	6	124	189	4	137	198
Recall	88.57%	72.56%	73.32%	86.89%	76.37%	78.57%	86.54%	71.69%	75.48%
Precision	91.18%	86.90%	87.05%	89.83%	83.83%	87.48%	91.84%	88.19%	88.62%

## Data Availability

The data for this study come from two main sources: 1. Due to the confidentiality agreement, the first dataset, of electric tricycles, is not available. This dataset consists of actual traffic images from the project team and cannot be shared. 2. The second source contains publicly available data in public repositories: COCO2017.
